# Role of ghrelin in promoting catch-up growth and maintaining metabolic homeostasis in small-for-gestational-age infants

**DOI:** 10.3389/fped.2024.1395571

**Published:** 2024-06-06

**Authors:** Li Zhang, Jingfei Liu, Dianyong Gao, Dong Li

**Affiliations:** ^1^ Department of Pediatrics, The Second Affiliated Hospital of Dalian Medical University, Dalian, China; ^2^ Department of Neonatology, Dalian Women and Children’s Medical Group, Dalian, China; ^3^ Department of Orthopedics, Lushunkou District People’s Hospital, Dalian, China; ^4^ Department of Neonatology, The First Affiliated Hospital of Dalian Medical University, Dalian, China

**Keywords:** ghrelin, metabolic homeostasis, catch-up growth, metabolic syndrome, small-for-gestational age

## Abstract

Small-for-gestational age (SGA) has been a great concern in the perinatal period as it leads to adverse perinatal outcomes and increased neonatal morbidity and mortality, has an impact on long-term health outcomes, and increases the risk of metabolic disorders, cardiovascular, and endocrine diseases in adulthood. As an endogenous ligand of the growth hormone secretagotor (GHS-R), ghrelin may play an important role in regulating growth and energy metabolic homeostasis from fetal to adult life. We reviewed the role of ghrelin in catch-up growth and energy metabolism of SGA in recent years. In addition to promoting SGA catch-up growth, ghrelin may also participate in SGA energy metabolism and maintain metabolic homeostasis. The causes of small gestational age infants are very complex and may be related to a variety of metabolic pathway disorders. The related signaling pathways regulated by ghrelin may help to identify high-risk groups of SGA metabolic disorders and formulate targeted interventions to prevent the occurrence of adult dwarfism, insulin resistance-related metabolic syndrome and other diseases.

## 1 Introduction

Small for gestational age (SGA) is a concern during the perinatal period. If the birth weight and/or birth length of a newborn is <2 standard deviations of the mean weight and length for the same gestational age, a clinical diagnosis of SGA is made (1). SGA is a syndrome associated with several factors, maternal diseases such as poor nutritional intake, endocrine diseases, drug use, lifestyle habits, genetic factors (2, 3), congenital infections of the fetus, chromosomal abnormalities, and genetic defect, etc. (4) may interfere with growth potential and affect the birth weight and length of the newborn. However, the pathogenic causes of SGA have not been completely elucidated. The incidence of SGA varies significantly in different regions, ranging from 7% in industrialized countries to 41.5% in South Asian countries (5, 6). On average, 16% of newborns have SGA globally. SGA not only leads to adverse perinatal outcomes, which increases the risk of neonatal morbidity and mortality, but also affects long-term health outcomes, which increases the incidence of short stature in adults. Simultaneously, the risks of future insulin resistance, lipid metabolism disorders, thyroid dysfunction, diabetes, coronary heart disease, cancer, and other diseases increase. Meanwhile, SGA might be associated with long-term neurological damage (7). Previous studies on the factors associated with the regulation of SGA growth and energy metabolism homeostasis can help prevent metabolic diseases in adulthood, which is a global public health issue.

Fetal growth is a complex process regulated by different factors. Regulation of fetal programing by the growth hormone–insulin-like growth factor (GH-IGF) axis has been proposed as the mechanism that could explain the link between low birth weight and adult disease. The GH receptor (GHR) mediates the effect of GH on linear growth and metabolism (8–10). Ghrelin—which is an endogenous ligand of the growth hormone secretagogue receptor (GHS-R)—promotes GH release, is involved in prenatal and postnatal growth, and is a possible predictor of catch-up growth in neonates and infants. SGA neonates with high ghrelin concentrations have a better catch-up growth (11). The ghrelin concentration of adolescent children born with SGA who completed catch-up growth was higher than that of adolescent children born with SGA who did not complete catch-up growth and short children with appropriate for gestational age (AGA) (controls). Moreover, previous studies have found that ghrelin concentration is negatively correlated with body mass index and insulin levels. Further, ghrelin may participate in energy metabolism while promoting growth and reducing the development of obesity and insulin resistance (12). In recent years, acyl-ghrelin (AG) has been playing a unique role in energy metabolism, which affects various systems in the body and is an important target in treating various diseases, including obesity, metabolic disorders, stress and anxiety, and drug addiction (13, 14). Ghrelin has been shown to regulate growth and energy metabolism homeostasis (15). Similarly, growth and metabolic disorders are also problems that SGA children may face. Does ghrelin participate in the pathogenesis of SGA, and what role does it play in the growth and metabolism of SGA? These are issues that we would like to explore. Therefore, this review aimed to assess the recent research progress on the association between ghrelin and SGA.

## 2 Expression, secretion, and metabolism of ghrelin

**Figure 1:**
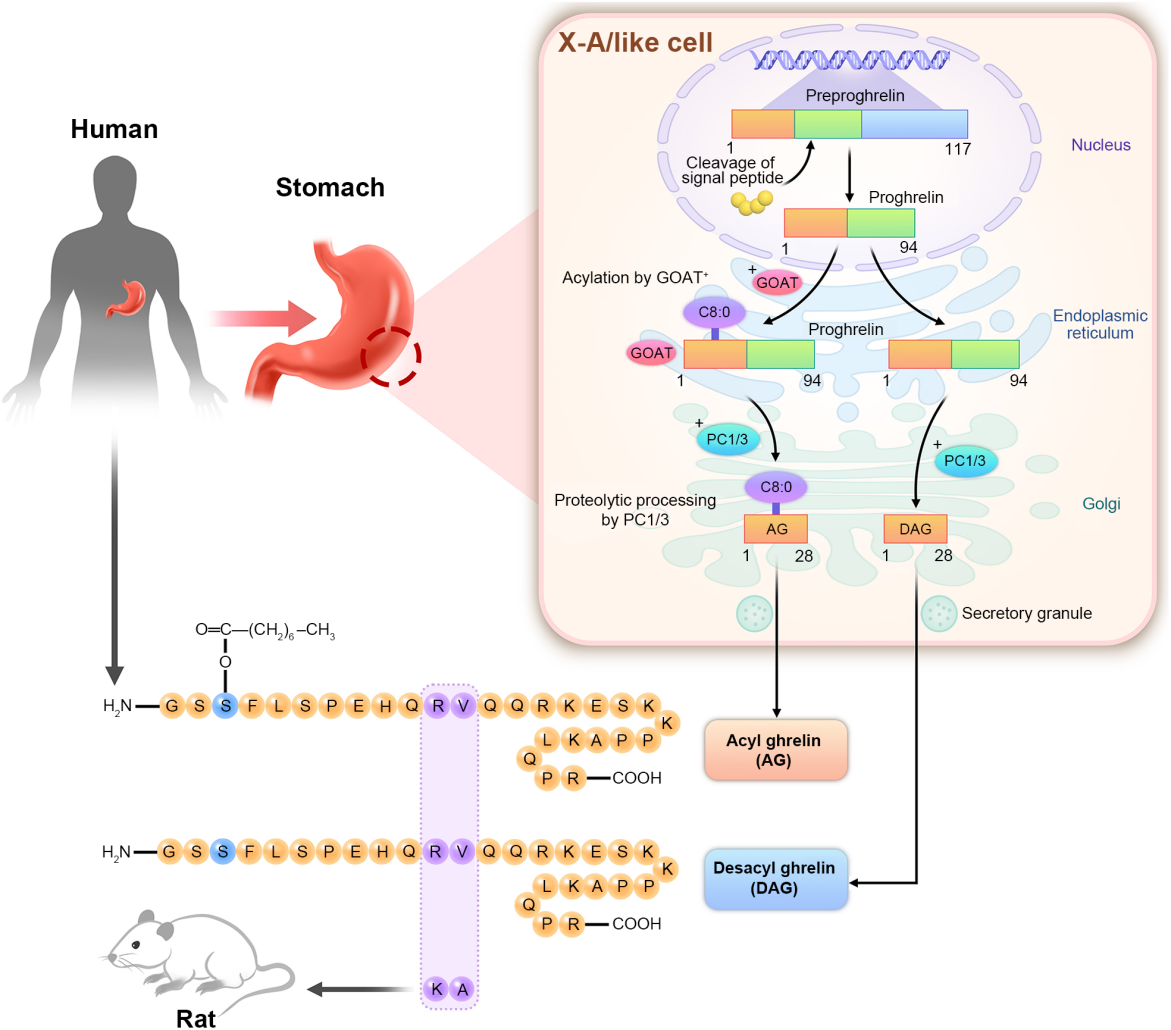
Structure diagram of AG and DAG. The human ghrelin gene is located on chromosome 3p25–26 and in the X/A-like cells of the gastric fundus. The ghrelin gene is translated into the 117-amino acid preproghrelin. Signal peptide cleaved the ghrelin precursor into 94-amino acid proghrelin. Serine was the third amino acid of the ghrelin precursor. In the endoplasmic reticulum, it is acetylated by O-acyltransferase (GOAT) to form acylated proghrelin, which is then transported to the Golgi apparatus. The cleavage of prohormone convertase1/3 (PC1/3) forms acylated ghrelin (AG) with 28 amino acids. The precursors of ghrelin that are not caplyacylated in the endoplasmic reticulum are directly cleaved by PC1/3 in the Golgi apparatus to produce des-acylated ghrelin (DAG). Human ghrelin and rat ghrelin have a high homology. Only 11 and 12 amino acids are different.

Ghrelin is a 28-amino acid polypeptide found in the X/A-like cells of rats and the human stomach based on immunohistochemistry performed in 1999 by Kojima. It is expressed in a small amount in the pancreas, thyroid, kidneys, lungs, placenta, and other tissues (16). Ghrelin is a natural endogenous ligand of GHS-R type 1a. Ghrelin promotes GH release and is the only orexin hormone produced by peripheral organs (17). They are divided into AG and desacyl-ghrelin (DAG) according to whether they are octylized by O-acyltransferase in the Endoplasmic Reticulum (ER) (16). As show in Figure 1, modified by n-octanoic acid is essential for the binding and activation of AG to GHS-R1a. Previous studies have revealed that DAG could not bind to GHS-R1a due to the lack of the n-octanoic acid modification. Hence, the corresponding receptor could not be activated, and only AG was biologically active (18). However, further research has increasingly recognized the active hormonal role of DAG, although is binding receptor is yet to be identified, and DAG affects various systems under different physiological and pathological conditions (19).

Ghrelin is secreted by X/A-like cells in the fundus of the stomach, circulated in blood, and transmitted through the vagus nerve to bindwith the GH-secreting receptor 1a (GHS-R1a). Meanwhile, GHS-Rla can be transported to gastric cells through vagus nerve transmission, binding to AG, inhibiting the electrical activity of labyrinths, and acting on central neurons. After binding to GHS-R1a, AG effectively activated the Hypothalamic arcuate nucleus (ARC) expresses the appetite promoting hormone Neuropeptide Y (NPY) and Agouti-related protein (AgRP), and these neurons signal to Ventromedial nucleus of the hypothalamus (VMH) and Paraventricular nucleus (PVN) through synapses to stimulate food intake, and stimulates GH release by acting on GHRH neurons or by directly activating somatotrophs in the anterior pituitary. The AG-GHS-Rla axis plays several biological roles by signal transmission through synapses. However, the signaling pathways through which DAG acts remain unclear (18).

Similar to other endocrine hormones—such as melatonin and GH—ghrelin is secreted in pulses in the human body and is influenced by the endogenous circadian rhythm, with the peak secretion at night (20). Since a positive correlation between nocturnal ghrelin and nocturnal GH secretion was noted, it appears that nocturnal measurements better reflect the pool of ghrelin responsible for stimulation of GH and IGF-I secretion (21). Moreover, the levels of ghrelin fluctuate with eating. The plasma concentrations of total ghrelin and AG increase before meals, decrease rapidly after meals, and gradually increase again until the next meal (22). The duration and extent of ghrelin reduction after a meal is related to the total calories and types of nutrients consumed, with carbohydrates and protein having the greatest impact. Insulin and glucose comprise the main determinants of ghrelin secretion. Insulin inhibits ghrelin secretion through the phosphatidylinositol 3-kinase/protein kinase B pathway. Moreover, age and sex may affect ghrelin secretion. In adults, the DAG levels are higher in women than in men (23), although other studies demonstrated similar total ghrelin levels between male and female newborns (24, 25).

In recent years, the brain–intestinal peptide ghrelin has attracted increasing attention because of its role in maintaining energy metabolism homeostasis. It can effectively maintain body metabolism and energy balance, regulate blood glucose levels and fat metabolism, and prevent fatal hypoglycemia while fasting. It also improve memory, prevent anxiety and depression, and protect metabolic organs (such as the liver), adipose tissue, skeletal muscle, and myocardium from stressful conditions (such as asphyxia, hypoxia, and burn) and inflammation. Furthermore, it plays a key regulatory role in maintaining metabolic energy homeostasis (26).

Based on these physiological effects of ghrelin, the relationship between ghrelin and SGA in growth and metabolism has gradually attracted attention, and an increasing number of studies have distinguished between AG and DAG. In neonates, DAG appeared to be higher in SGA than in AGA, but the AG level did not differ (27). However, to date, the roles of AG and DAG in neonates and children—particularly at birth—remain unclear and poorly studied.

## 3 Role of ghrelin in fetal development

Maternal ghrelin plays an important role in fetal growth and development. Previous studies have found that maternal exogenous ghrelin supplementation can increase fetal birth weight (28), and ghrelin-resistant mothers deliver neonates with a low birth weight (29). Based on these findings, at least part of the fetal ghrelin comes from the maternal circulation. Clinical studies have found that the serum ghrelin level during pregnancy peaks at the second trimester, and it is at its lowest during the third trimester. These findings are consistent with the development of maternal weight gain and insulin resistance (30). Maternal AG levels are positively correlated with newborn waist circumference at birth. Further, newborn waist circumference measured at birth is an indicator of liver volume and visceral fat, reflecting the energy deposition of newborns (31). The positive correlation between maternal ghrelin levels and neonatal waist circumference during the second and third trimesters indicate that ghrelin can be involved in the energy balance of the fetus, regulating fat-energy deposition in newborns. In addition, it can be a predictor of the future growth and metabolic health of the newborn. Infer from this, ghrelin may plays a role in maternal energy regulation, and it is related to the nutritional supply of the fetus.

Several studies have analyzed the presence of ghrelin in the cord blood. Ghrelin has been detected in the cord blood of fetuses at 30 weeks of gestation (32). Based on an assessment using newborn cord blood samples, low-birth-weight newborns had higher ghrelin levels in their cord blood than high-birth-weight newborns. Fetal ghrelin levels may reflect energy supply in the womb. The lack of correlation between placental ghrelin expression and cord blood ghrelin level shows that placental and fetal ghrelin can be produced separately. Further, ghrelin in the cord blood mainly comes from the fetus (33). In another study, the level of total ghrelin in the umbilical vein serum samples was found significantly lower than that in the umbilical artery samples; this meant that ghrelin in cord blood primarily comes from the fetus (34).

The association between cord blood ghrelin levels and newborn birth weight is controversial. Previous studies have reported negative associations of serum ghrelin with AG and DAG levels (25, 35, 36). Small-for-gestational-age infants have higher ghrelin levels, with a gradual increase in total ghrelin levels within the first few days after birth that is significantly higher than the levels detected in the cord blood (37). The cord blood total ghrelin level of SGA preterm infants is higher than that of AGA preterm infants (38, 39). One study suggested that neonatalcord blood ghrelin concentration might be a birth weight determinant (40). In addition, the concentration of AG is negatively correlated with neonatal head circumference, abdominal circumference, and thigh circumference (34). Ghrelin may plays a physiological role in regulating growth in the early stage of life. However, some studies have different findings. That is, no difference was observed in the serum ghrelin levels between full-term and preterm infants and between AGA and large for gestational age (LGA) infants (19, 41, 42). Moreover, SGA neonates have lower cord blood ghrelin levels (43).

The conflicting results could be attributed to the detection methods used. For example, AG and DAG could have been detected individually, and different methods were utilized to stabilize AG in the samples. AG in the plasma is easily converted to DAG. Therefore, the method used to stabilize AG during the experiment is important in obtaining accurate experimental findings (44). In recent years, an increasing number of studies have conducted a differential analysis of AG and DAG to distinguish their physiological effects. Some studies have found that the cord blood DAG concentration of SGA neonates is significantly higher than that of AGA neonates. Moreover, DAG is negatively correlated with birth weight and placental weight. Hence, DAG plays a more important role in birth weight than AG (45). The difference between AG and DAG should be analyzed to distinguish their physiological effects. In addition, there can be a compensatory mechanism for the negative energy balance in SGA fetuses, and high ghrelin levels can be a manifestation of an adaptive response. The correlation between umbilical cord blood ghrelin concentration and fetal growth should be validated.

## 4 Association between ghrelin and catch-up growth in SGA infants

In the early postnatal period, infants with SGA usually have a faster growth rate and weight increase rate than those with AGA, a condition known as catch-up growth (CUG), which refers to height growth that exceeds the upper end of the normal range for the same age for at least 1 year after a short period of growth inhibition (46). It can be continuous or discontinuous. If the final adult height is within the target range, CUG may be considered complete. Previous research has proposed two types of neuroendocrine and growth plate models for CUG (47). Another study has reported that 69%–82.2% of SGA newborns complete CUG at the age of 1 year, and the completion rate at the age of 2 years is approximately 87.4%–96.6%, the catch-up growth in a premature child born with SGA may last until 4 years of age (48).

Nevertheless, the growth trajectory of height, weight, and head circumference of some SGA newborns after birth is significantly lower than that of AGA newborns. Moreover, the three index of some SGA newborns within the first 3 years after birth are still significantly lower than those of AGA infants, which eventually leads to lifelong height and short stature in adulthood, with the proportion reaching 10% (49, 50). Similarly, the GH secretion of adult born with SGA based on the growth hormone stimulation tests is normal. However, their insulin-like growth factor type I (IGF-I) concentrations are typically low, and their final height ratio is approximately 1.0 standard deviation below the target height (TH) (51). For those with SGA who cannot complete CUG, GH supplementation to improve short stature has been included in the treatment guidelines.

Approximately 10% of children with SGA do not complete CUG, and the mechanism of which is unknown. Some studies have revealed that it may be related to GHR or IGF-1R gene mutation (52), and current research has focused on the specific factors regulating CUG. In vivo, ghrelin and growth hormone-releasing hormone work together to stimulate growth hormone secretion (53). AG acts on the GSHR-1a receptor of pituitary growth hormone-secreting cells and activates phospholipase C. It results in the production of inositol 1,4,5-triphosphate and diacylglycerol, which increase intracellular Ga2+ and promote GH release (54). Ghrelin may be a strong predictor of CUG in newborns with SG. One week after birth, SGA infants presented with a significant increase in total ghrelin levels (39). Higher total ghrelin levels in newborns with SGA at birth, which remain elevated at 3 months of age, are associated with anthropometric markers at birth and early postnatal growth (55). In identical twins with SGA, neonates with high ghrelin concentrations completed CUG and maintained high ghrelin concentrations in the first year after birth, a rapid CUG rate in both height and weight was detected. Compared with infants who have not experienced CUG, SGA infants had higher postprandial ghrelin concentrations within the first year after birth (11). These clinical studies suggested that high ghrelin levels promote increased appetite in these infants and ensure nutrient intake, resulting in rapid CUG.

## 5 Maintenance of SGA metabolic homeostasis by ghrelin

Previous studies have commonly revealed that ghrelin plays a role in promoting CUG in SGA. But he metabolic aspects of SGA have not been reported. However, individuals with SGA who presented with a rapid increase in height and weight at the start of birth have a higher incidence of metabolic disorders (56). What role does ghrelin play as a somatostatin-releasing peptide in promoting CUG and metabolic disorders in individuals with SGA? It is worthy for clinical attention and further research.

Children born with SGA are at significantly increased risk of hypertension (57, 58), insulin resistance (59), dyslipidemia, and nonalcoholic fatty liver disease (60) during adolescence, which lead to type 2 diabetes (DM2), central obesity, and cardiovascular disease in adulthood (61). This mechanism between fetal growth restriction and later metabolic disease is currently explained by the “thrifty phenotype hypothesis” in most studies (62). To adapt to the malnourished intrauterine environment, the developing fetus may appear to dysregulation of the neuroimmune–endocrine axis (63), and this adaptation process in fetal life results in changes in insulin signaling pathways and glucose metabolism (64). Intrauterine malnutrition induces fetal programming, leading to rapid CUG and insulin resistance in individuals born with SGA and who are at risk of glucose and fat metabolism disorders and adverse metabolic diseases (59, 65). Further, disturbances in the hypothalamic-pituitary-thyroid axis and hypothalamic–pituitary adrenal (HPA) axis may be involved (66).

The increased risk of metabolic disorders and cardiovascular disease with SGA is present not only in adulthood, but also in childhood. SGA newborns have metabolic disorders. The incidence rate of hypoglycemia in neonates with SGAs is 15%–36% (67). This condition is caused by low hepatic glycogen and fat storage, inefficient production of glucose via the gluconeogenic pathway, higher energy requirements, increased insulin sensitivity, and lack of an antiregulatory hormonal response (68). Simultaneously, some studies have found a temporal association between low birth weight and insulin sensitivity reduction (69). Blood lipid levels in SGA infants are different from those in AGA newborns. The results of the neonatal vein blood test performed within 72 h after birth have shown that the levels of triglyceride, total cholesterol, and low-density lipoprotein cholesterol in SGA newborns are higher than those in AGA newborns. The levels of total cholesterol, low-density lipoprotein cholesterol, high-density lipoprotein cholesterol, and apolipoprotein A in newborns with a birth body mass index of <10th percentile were lower than those with a birth body mass index of ≥10th percentile. Furthermore, newborns with a birth body mass index of <10th percentile had higher levels of triglyceride and apolipoprotein B than those with a birth body mass index of the ≥10th percentile. The greater degree of fetal growth restriction, the level of lipid metabolism disorder is more evident (70).

In childhood, those who are born with SGA have higher fasting blood glucose levels than children with normal weight at birth, and higher fasting blood glucose levels may be precursors to hyperinsulinemia, insulin resistance, and type 2 diabetes (70). Children with SGA are at a significantly increased risk for both type 2 diabetes and insulin resistance (62), which are more evident in cases of rapid weight gain in infancy (71). The redistribution of weight gain promotes the accumulation of abdominal fat, which occurs primarily between the ages of 2 and 4 years. At 4 years of age, children with SGA have a higher fat mass, insulin resistance, and proinflammatory parameters (72). Low-birth-weight children have an increased risk of hypercholesterolemia. Children born with SGA have increased abdominal fat in preschool age, and obesity significantly promotes insulin resistance (67). Meanwhile, SGA children with poor CUG in height may be at the highest risk for hypercholesterolemiain childhood (73).

In contrast, other studies have revealed that rapid CUG and insulin resistance could not completely explain the association between SGA and metabolic disorders (63). By most accounts, metabolic disorder is caused by an adverse intrauterine environment that may trigger epigenetic regulation and impaired liver growth. If the body mass index of children born with SGA is within the normal range, their insulin sensitivity does not change, and CUG does not affect insulin resistance in participants born with SGA (74). Compared born with SGA, obesity resulting from excessive CUG later in life is more likely to lead to insulin resistance. Therefore, a dynamic change in individual obesity is involved in the long-term metabolic outcome of SGA (75). Although SGA is associated with adverse metabolic characteristics in overweight or obese children but its effect is extremely small compared with the severity of obesity (66), and these findings underscore the importance of weight management. Obese children and adolescents born with SGA are more likely to have metabolic risk factors compared with those born with AGA (56). Therefore, it is important to clear the mechanisms of fetal growth and metabolic patterns that lead to SGA.

The mechanisms related to metabolic disorders in individuals born with SGA have been the focus of research. Imbalances in ghrelin, adiponectin, and leptin may be a risk factor for fetal growth retardation and future metabolic diseases (38). The central ghrelin signaling system has a powerful appetite stimulating effect. If ghrelin is delivered to most brain regions where GHS-R is located, it can drive the eating response (76). For a long time, it was considered obesity-promoting. However, obesity is not caused by high ghrelin levels (77). Diet-induced obesity impairs the ghrelin signaling pathway, leading to ghrelin resistance (78). Rather than causing overeating, ghrelin promotes appetite, food preference selection, and food reward (79). In addition, an increasing number of studies have found that ghrelin maintains the role of metabolic homeostasis.

In animal experimental studies, daily peripheral injection of ghrelin in mice can lead to increased body fat and weight, the mechanism leading to this result is not attributed to increased food intake with ghrelin supplementation. However, it can be related to decreased fat usage (80). AG changes fatty acid metabolism via the intracellular signaling pathways in the hypothalamus and regulates central and peripheral lipid metabolism (81, 82). In addition, ghrelin reduces hepatocyte lipid toxicity, mitochondrial dysfunction, endoplasmic reticulum stress, programmed cell death, the reversibility of the proinflammatory phenotype of Kupffer cells, and hepatic stellate cell inactivation via autophagy and fatty acid β-oxidation (83). The metabolic and inflammatory pathways regulated by ghrelin in the liver support its potential as a therapeutic target for the prevention of nonalcoholic fatty liver disease in patients with metabolic disorders. The ghrelin–GHS–R1a axis also regulates glucose homeostasis via a central mechanism. Ghrelin prevents life-threatening hypoglycemia during fasting and energy restriction (84). A recent clinical study found that infants with very low birth weight (VLBW) appeared to have a hormone profile consistent with insulin resistance, which may be associated with significantly elevated concentrations of ghrelin (85). As a negative regulator of insulin secretion, AG is dependent on GHS-R1a signaling in cells and interacts with somatostatin receptor subtype 5 to stimulate the secretion of glucagon by islet alpha cells and regulate glucose metabolism (86).

Current studies have shown that the ghrelin levels peak early after birth, until GH begins its function in regulating nutrient intake and growth (19). Normal-weight infants have high ghrelin levels, while infants with obesity or those whose growth accelerates within the first year of life have low ghrelin levels (87, 88). As a metabolic signal, ghrelin may play an important effect on regulating energy balance during early life growth and development. The role of ghrelin as a predictor of or intervention target for SGA metabolic disorders must be further explored.

## 6 Conclusions

In addition to promoting appetite, ghrelin has been found maintain growth hormone secretion and energy metabolism homeostasis. Previous studies mainly focused on the correlation between blood ghrelin concentration and birth weight, CUG, and adult short stature. Whether intrauterine malnutrition or rapid CUG is the main cause of metabolic disorders in individuals born with SGA? Dose ghrelin reduce or increase the risk of metabolic disease? Can ghrelin be used as a biomarker of metabolic health in early life? At present, there is no clear conclusion, and the mechanism of ultimate height reduction and metabolic complications in individuals born with SGA should be further validated.

Genetic and metabolic factors that contribute to SGA are complex, and they involve dysregulation of multiple metabolic pathways, which are not well understood. The function of ghrelin in SGA status and metabolism should not be simply determined based on ghrelin levels. Further research should focus on the relevant signaling pathways regulated by ghrelin, which can help identify high-risk groups with SGA-related metabolic disorders and develop targeted interventions to prevent the occurrence of diseases such as dwarfism and insulin resistance-related metabolic syndrome in adulthood.

## References

[1] Hokken-Koelega ACS van der Steen M Boguszewski MCS Cianfarani S Dahlgren J Horikawa R International consensus guideline on small for gestational age (SGA): etiology and management from infancy to early adulthood. Endocr Rev. (2023) 44:539–65. 10.1210/endrev/bnad002 36635911 PMC10166266

[2] Del Gobbo GF Yin Y Choufani S. Genomic imbalances in the placenta are associated with poor fetal growth. Pak J Med Sci. (2022) 38:219–26. 10.12669/pjms.38.1.4396 33413077 PMC7792164

[3] Andersson NW Skov L Andersen JT. Evaluation of topical corticosteroid use in pregnancy and risk of newborns being small for gestational age and having low birth weight. JAMA Dermatol. (2021) 157:788–95. 10.1001/jamadermatol.2021.1090 33950165 PMC8100914

[4] Leite DFB Morillon AC Melo Júnior EF Souza RT McCarthy FP Khashan A Examining the predictive accuracy of metabolomics for small-for-gestational-age babies: a systematic review. BMJ Open (2019) 9:e031238. 10.1136/bmjopen-2019-031238 31401613 PMC6701563

[5] Lee ACC Katz J Blencowe H Cousens S Kozuki N Vogel JP National and regional estimates of term and preterm babies born small for gestational age in 138 low-income and middle-income countries in 2010. Lancet Glob Health. (2013) 1:e26–36. 10.1016/S2214-109X(13)70006-8 25103583 PMC4221634

[6] Tudehope D Vento M Bhutta Z Pachi P. Nutritional requirements and feeding recommendations for small for gestational age infants. J Pediatr. (2013) 162(suppl):S81–9. 10.1016/j.jpeds.2012.11.057 23445853

[7] Motte-Signoret E Shankar-Aguilera S Brailly-Tabard S Soreze Y Dell Orto V Ben Ammar R Small for gestational age preterm neonates exhibit defective GH/IGF1 signaling pathway. Front Pediatr. (2021) 9:711400. 10.3389/fped.2021.711400 34447729 PMC8382944

[8] Kaur H Muhlhausler BS Roberts CT Gatford KL. The growth hormone-insulin like growth factor axis in pregnancy. J Endocrinol. (2021) 1:JOE-21-0087.R1. 10.1530/JOE-21-0087 34479185

[9] Perez Garrido N Pujana M Berger M Ramírez P Guercio G Belgorosky A Growth hormone receptor gene polymorphism. Spontaneous catch up growth in small for gestational age patients. Medicina (B Aires). (2021) 81:574–80. PMID: 34453799

[10] Holt RIG. Fetal programming of the growth hormone-insulin-like growth factor axisl. Trends Endocrinol Metab. (2002) 13:392–7. 10.1016/s1043-2760(02)00697-5 12367821

[11] Gohlke BC Huber A Hecher K Fimmers R Bartmann P Roth CL. Fetal insulin-like growth factor (IGF)-I, IGF-II, and ghrelin in association with birth weight and postnatal growth in monozygotic twins with discordant growth. J Clin Endocrinol Metab. (2005) 90:2270–4. 10.1210/jc.2004-1192 15687342

[12] Stawerska R Szałapska M Hilczer M Lewiński A. Ghrelin, insulin-like growth factor I and adipocytokines concentrations in born small for gestational age prepubertal children after the catch-up growth. J Pediatr Endocrinol Metab. (2016) 29:939–45. 10.1515/jpem-2015-0463 27269893

[13] Pietrzak M Yngve A Hamilton JP Asratian A Gauffin E Löfberg A Ghrelin decreases sensitivity to negative feedback and increases prediction-error related caudate activity in humans: a randomized controlled trial. Neuropsychopharmacology. (2024) 49:1042–9. 10.1038/s41386-024-01821-6 38409282 PMC11039644

[14] Wang Y Guo S Zhuang Y Yun Y Xu P He X Molecular recognition of an acyl-peptide hormone and activation of ghrelin receptor. Nat Commun. (2021) 12:5064. 10.1038/s41467-021-25364-2 34417468 PMC8379176

[15] Rouault AAJ Rosselli-Murai LK Hernandez CC Gimenez LE Tall GG Sebag JA. The GPCR accessory protein MRAP2 regulates both biased signaling and constitutive activity of the ghrelin receptor GHSR1a. Sci Signal. (2020) 13:eaax4569. 10.1126/scisignal.aax4569 31911434 PMC7291826

[16] Kojima M Kangawa K. Ghrelin: more than endogenous growth hormone secretagogue. Ann N Y Acad Sci. (2010) 1200:140–8. 10.1111/j.1749-6632.2010.05516.x 20633142

[17] Ogawa S Liu X Shepherd BS Parhar IS. Ghrelin stimulates growth hormone release from the pituitary via hypothalamic growth hormone-releasing hormone neurons in the cichlid, Oreochromis niloticus. Cell Tissue Res. (2018) 374:349–65. 10.1007/s00441-018-2870-6 29934855

[18] Yanagi S Sato T Kangawa K Nakazato M. The homeostatic force of ghrelin. Cell Metab. (2018) 27:786–804. 10.1016/j.cmet.2018.02.008 29576534

[19] González-Domínguez MI Lazo-de-la-Vega-Monroy ML Zaina S Sabanero M Daza-Benítez L Malacara JM Association of cord blood des-acyl ghrelin with birth weight, and placental GHS-R1 receptor expression in SGA, AGA, and LGA newborns. Endocrine. (2016) 53:182–91. 10.1007/s12020-015-0833-1 26754660

[20] Qian J Morris CJ Caputo R Garaulet M Scheer FAJL. Ghrelin is impacted by the endogenous circadian system and by circadian misalignment in humans. Int J Obes. (2019) 43:1644–9. 10.1038/s41366-018-0208-9 PMC6424662 30232416

[21] Stawerska R Kolasa-Kicińska M Łupińska A Hilczer M Lewiński A. Comparison of nocturnal and morning ghrelin concentration in children with growth hormone deficiency and with idiopathic short stature. Chronobiol Int. (2020) 37:1629–35. 10.1080/07420528.2020.1797765 32779492

[22] Di Bonaventura EM Botticelli L Bello FD. Assessing the role of ghrelin and the enzyme ghrelin O-acyltransferase (GOAT) system in food reward, food motivation, and binge eating behavior. Pharmacol Res. (2021) 8:23. 10.1016/j.phrs.2021.105847 34438062

[23] Anderson KC Hasan F Grammer EE Kranz S. Endogenous ghrelin levels and perception of hunger: a systematic review and meta-analysis. Adv Nutr. (2023) 14:1226–36. 10.1016/j.advnut.2023.07.011 37536563 PMC10509419

[24] Pirazzoli P Lanari M Zucchini S Gennari M Pagotto U De Iasio RCicognani A Active and total ghrelin concentrations in the newborn. J Pediatr Endocrinol Metab. (2005) 18:379–84. 10.1515/jpem.2005.18.4.379 15844472

[25] Warchoł M Wojciechowska M Kupsz J Sot-Szewczyk MH Michalak M Kołodziejski P Association of cord blood ghrelin, leptin and insulin concentrations in term newborns with anthropometric parameters at birth. J Pediatr Endocrinol Metab. (2018) 31:151–7. 10.1515/jpem-2017-0285 29320365

[26] Zhang L Li D. Research progress on the role of ghrelin in maintaining metabolism and energy homeostasis. Chin J Child Health Care. (2022) 30:994–9. 10.11852/zgetbjzz2022-0327

[27] Bellone S Prodam F Savastio S Avanzo D Pagani A Trovato L Acylated/unacylated ghrelin ratio in cord blood: correlation with anthropometric and metabolic parameters and pediatric lifespan comparison. Eur J Endocrinol. (2012) 166:115–20. 10.1530/EJE-11-0346 22004908

[28] Nakahara K Nakagawa M Baba Y Sato M Toshinai K Date Y Maternal ghrelin plays an important role in rat fetal development during pregnancy. Endocrinology. (2006) 147:1333–42. 10.1210/en.2005-0708 16339208

[29] Schalla MA Stengel A. The role of the gastric hormones ghrelin and nesfatin-1 in reproduction. Int J Mol Sci. (2021) 22:11059. 10.3390/ijms222011059 34681721 PMC8539660

[30] Garcés MF Buell-Acosta JD Ángel-Müller E Parada-Baños AJ Acosta-Alvarez J Saavedra-López HF Study of the ghrelin/LEAP-2 ratio in humans and rats during different phases of pregnancy. Int J Mol Sci. (2022) 23:9514. 10.3390/ijms23179514 36076912 PMC9455743

[31] Valsamakis G Papatheodorou DC Naoum A Margeli A Papassotiriou I Kapantais E Neonatal birth waist is positively predicted by second trimester maternal active ghrelin, a pro-appetite hormone, and negatively associated with third trimester maternal leptin, a pro-satiety hormone. Early Hum Dev. (2014) 90:487–92. 10.1016/j.earlhumdev.2014.07.001 25051539

[32] Soriano-Guillén L Barrios V Chowen JA Sánchez I Vila S Quero J Ghrelin levels from fetal life through early adulthood: relationship with endocrine and metabolic and anthropometric measures. J Pediatr. (2004) 144:30–5. 10.1016/j.jpeds.2003.08.050 14722515

[33] Allbrand M Åman J Lodefalk M. Placental ghrelin and leptin expression and cord blood ghrelin, adiponectin, leptin, and C-peptide levels in severe maternal obesity. J Matern Fetal Neonatal Med. (2018) 31:2839–46. 10.1080/14767058.2017.1358262 28783996

[34] Fuglsang J Sandager P Møller N Fisker S Frystyk J Ovesen P. Peripartum maternal and foetal ghrelin, growth hormones, IGFs and insulin interrelations. Clin Endocrinol. (2006) 64:502–9. 10.1111/j.1365-2265.2006.02498.x 16649967

[35] He H Zhu W-T Nuyt AM Marc I Julien P Huang R Cord blood IGF-I, proinsulin, leptin, HMW adiponectin, and ghrelin in short or skinny small-for-gestational-age infants. J Clin Endocrinol Metab. (2021) 106:e3049–57. 10.1210/clinem/dgab178 33738477

[36] Méndez-Ramírez F Barbosa-Sabanero G Romero-Gutiérrez G Malacara JM. Ghrelin in small-for-gestational age (SGA) newborn babies: a cross-sectional study. Clin Endocrinol. (2009) 70:41–6. 10.1111/j.1365-2265.2008.03278.x 18419783

[37] Sahin H Erener T Erginoz E Vural M Ilikkan B Kavuncuoglu S The relationship of active ghrelin levels and intrauterine growth in preterm infants. Eur J Endocrinol. (2012) 166:399–405. 10.1530/EJE-11-0607 22143318

[38] Han L Li B Xu X Liu S Li Z Li M Umbilical cord blood adiponectin, leptin, insulin, and ghrelin in premature infants and their association with birth outcomes. Front Endocrinol. (2021) 2:738964. 10.3389/fendo.2021.738964 PMC8515017 34659122

[39] Magalhães ESDS Méio MDBB Peixoto-Filho FM Gonzalez S da Costa ACC Moreira MEL. Pregnancy-induced hypertension, preterm birth, and cord blood adipokine levels. Eur J Pediatr. (2020) 179:1239–46. 10.1007/s00431-020-03586-8 32062709

[40] Farquhar J Heiman M Wong ACK Wach R Chessex P Chanoine JP. Elevated umbilical cord ghrelin concentrations in small for gestational age neonates. J Clin Endocrinol Metab. (2003) 88:4324–7. 10.1210/jc.2003-030265 12970305

[41] Özdemir ZC Akşit MA. The association of ghrelin, leptin, and insulin levels in umbilical cord blood with fetal anthropometric measurements and glucose levels at birth. J Matern Fetal Neonatal Med. (2018) 33:1–151. 10.1080/14767058.2018.1520828 30185078

[42] Zhang S Zhai G Zhang J Zhou J Chen C. Ghrelin and obestatin plasma levels and ghrelin/obestatin prepropeptide gene polymorphisms in small for gestational age infants. J Int Med Res. (2014) 42:1232–42. 10.1177/0300060514533525 25223427

[43] Yalinbas EE Binay C Simsek E Aksit MA. The role of umbilical cord blood concentration of IGF-I, IGF-II, leptin, adiponectin, ghrelin, resistin, and visfatin in fetal growth. Am J Perinatol. (2019) 36:600–8. 10.1055/s-0038-1672141 30282106

[44] Alsaif M Pakseresht M Mackenzie ML Gaylinn B Thorner MO Freemark M Dietary macronutrient regulation of acyl and desacyl ghrelin concentrations in children with Prader-Willi syndrome (PWS). Clin Endocrinol. (2020) 93:579–89. 10.1111/cen.14279 32638409

[45] Bucur-Grosu ML Avasiloaiei A Moscalu M Dimitriu DC Păduraru L Stamatin M. DESACYLATED Ghrelin and leptin in the cord blood of SMALL-FOR-gestational-age newborns with intrauterine growth restriction. Acta Endocrinol. (2019) 15:305–10. 10.4183/aeb.2019.305 PMC6992402 32010348

[46] Halilagic A Moschonis G. The effect of growth rate during infancy on the risk of developing obesity in childhood: a systematic literature review. Nutrients. (2021) 13:3449. 10.3390/nu13103449 34684450 PMC8537274

[47] Gafni RI Baron J. Catch-up growth: possible mechanisms. Pediatr Nephrol. (2000) 14:616–9. 10.1007/s004670000338 10912529

[48] Campisi SC Carbone SE Zlotkin S. Catch-up growth in full-term small for gestational age infants: a systematic review. Adv Nutr. (2019) 10:104–11. 10.1093/advances/nmy091 30649167 PMC6370265

[49] Hendrix MLE van Kuijk SMJ El Bahaey SE Gerver WJM Feron FJM Kuin ME Postnatal growth during the first five years of life in SGA and AGA neonates with reduced fetal growth. Early Hum Dev. (2020) 151:105199. 10.1016/j.earlhumdev.2020.105199 33032049

[50] Jaquet D Collin D Lévy-Marchal C Czernichow P. Adult height distribution in subjects born small for gestational age. Horm Res. (2004) 62:92–6. 10.1159/000079709 15263821

[51] Putzker S Pozza SB Kugler K Schwarz HP Bonfig W. Insulin resistance in young adults born small for gestational age (SGA). J Pediatr Endocrinol Metab. (2014) 27:253–9. 10.1515/jpem-2013-0292 24152890

[52] Stróżewska W Durda-Masny M Szwed A. Mutations in GHR and IGF1R genes as a potential reason for the lack of catch-up growth in SGA children. Genes. (2022) 13:856. 10.3390/genes13050856 35627241 PMC9140854

[53] Liu H Sun D Myasnikov A Damian M Baneres JL Sun J Structural basis of human ghrelin receptor signaling by ghrelin and the synthetic agonist ibutamoren. Nat Commun. (2021) 12:6410. 10.1038/s41467-021-26735-5 34737341 PMC8568970

[54] Gupta D Patterson AM Osborne-Lawrence S Bookout AL Varshney S Shankar K Disrupting the ghrelin-growth hormone axis limits ghrelin’s orexigenic but not glucoregulatory actions. Mol Metab. (2021) 53:101258. 10.1016/j.molmet.2021.101258 34023483 PMC8203846

[55] Fidancı K Meral C Süleymanoğlu S Pirgon Ö Karademir F Aydınöz S Ghrelin levels and postnatal growth in healthy infants 0–3 months of age. J Clin Res Pediatr Endocrinol. (2010) 2:34–8. 10.4274/jcrpe.v2i1.34 21274334 PMC3005658

[56] Prinz N Putri RR Reinehr T Danielsson P Weghuber D Norman M The association between perinatal factors and cardiometabolic risk factors in children and adolescents with overweight or obesity: a retrospective two-cohort study. PLoS Med. (2023) 20:e1004165. 10.1371/journal.pmed.1004165 36638094 PMC9886302

[57] Yun M Wang X Fan L Yan Y Bazzano L He J Age-related suppression effect of current body weight on the association between birthweight and blood pressure: the Bogalusa heart study. Pediatr Obes. (2021) 16:e12716. 10.1111/ijpo.12716 32844607

[58] Das SK McIntyre HD Al Mamun A. Early life predictors of development of blood pressure from childhood to adulthood: evidence from a 30-year longitudinal birth cohort study. Atherosclerosis. (2020) 311:91–7. 10.1016/j.atherosclerosis.2020.09.001 32949948

[59] Martín-Calvo N Goni L Tur JA Martínez JA. Low birth weight and small for gestational age are associated with complications of childhood and adolescence obesity: systematic review and meta-analysis. Obes Rev. (2022) 23(1):e13380. 10.1111/obr.13380 34786817

[60] Nobili V Marcellini M Marchesini G Vanni E Manco M Villani A Intrauterine growth retardation, insulin resistance, and nonalcoholic fatty liver disease in children. Diabetes Care. (2007) 30:2638–40. 10.2337/dc07-0281 17536073

[61] Goedegebuure WJ Van der Steen M Smeets CCJ Kerkhof GF Hokken-Koelega ACS. SGA-born adults with postnatal catch-up have a persistently unfavourable metabolic health profile and increased adiposity at age 32 years. Eur J Endocrinol. (2022) 187:15–26. 10.1530/eje-21-1130 35521698

[62] Hales CN Barker DJ. The thrifty phenotype hypothesis. Br Med Bull. (2001) 60:5–20. 10.1093/bmb/60.1.5 11809615

[63] Bateson P Barker D Clutton-Brock T Deb D D’Udine B Foley RA Developmental plasticity and human health. Nature. (2004) 430:419–21. 10.1038/nature02725 15269759

[64] Cutfield W Ayyavoo A. The auxological and metabolic consequences for children born small for gestational age. Indian J Pediatr. (2021) 88:1235–40. 10.1007/s12098-021-03897-0 34405367

[65] Querter I Pauwels NS De Bruyne R Dupont E Verhelst X Devisscher L Maternal and perinatal risk factors for pediatric nonalcoholic fatty liver disease: a systematic review. Clin Gastroenterol Hepatol. (2022) 20:740–55. 10.1016/j.cgh.2021.04.014 33862225

[66] Blusková Z Koštálová L Celec P Vitáriušová E Pribilincová Z Maršálková M Evaluation of lipid and glucose metabolism and cortisol and thyroid hormone levels in obese appropriate for gestational age (AGA) born and non-obese small for gestational age (SGA) born prepubertal Slovak children. J Pediatr Endocrinol Metab. (2014) 27:693–9. 10.1515/jpem-2013-0334 24706427

[67] Bragg JJ Green R Holzman IR. Does early enteral feeding prevent hypoglycemia in small for gestational age neonates? J Neonatal Perinatal Med. (2013) 6:131–5. 10.3233/NPM-1366212 24246515

[68] Wang L-Y Wang L-Y Wang Y-L Ho C-H. Early neonatal hypoglycemia in term and late preterm small for gestational age newborns. Pediatr Neonatol. (2023) 64:538–46. 10.1016/j.pedneo.2022.09.021 36894475

[69] Wang X Cui Y Tong X Ye H Li S. Glucose and lipid metabolism in small-for-gestational-age infants at 72 h of age. J Clin Endocrinol Metab. (2007) 92:681–4. 10.1210/jc.2006-1281 17148563

[70] Kelishadi R Badiee Z Adeli K. Cord blood lipid profile and associated factors: baseline data of a birth cohort study. Paediatr Perinat Epidemiol. (2007) 21:518–24. 10.1111/j.1365-3016.2007.00870.x 17937737

[71] Stettler N Stallings VA Troxel AB Zhao J Schinnar R Nelson SE Weight gain in the first week of life and overweight in adulthood: a cohort study of European American subjects fed infant formula. Circulation. (2005) 111:1897–903. 10.1161/01.CIR.0000161797.67671.A7 15837942

[72] Ibáñez L Ong K Dunger DB de Zegher F. Early development of adiposity and insulin resistance after catch-up weight gain in small-for-gestational-age children. J Clin Endocrinol Metab. (2006) 91:2153–8. 10.1210/jc.2005-2778 16537681

[73] Tenhola S Martikainen A Rahiala E Herrgârd E Halonen P Voutilainen R. Serum lipid concentrations and growth characteristics in 12-year-old children born small for gestational age. Pediatr Res. (2000) 48:623–8. 10.1203/00006450-200011000-00012 11044482

[74] Deodati A Inzaghi E Cianfarani S. Epigenetics and in utero acquired predisposition to metabolic disease. Front Genet. (2019) 10:1270. 10.3389/fgene.2019.01270 32082357 PMC7000755

[75] Pham A Mitanchez D Forhan A Perin L Le Bouc Y Brioude F Low maternal DLK1 levels at 26 weeks is associated with small for gestational age at birth. Front Endocrinol. (2022) 13:836731. 10.3389/fendo.2022.836731 PMC8919710 35295988

[76] Zigman JM Jones JE Lee CE Saper CB Elmquist JK. Expression of ghrelin receptor mRNA in the rat and the mouse brain. J Comp Neurol. (2006) 494:528–48. 10.1002/cne.20823 16320257 PMC4524499

[77] Shiiya T Nakazato M Mizuta M Date Y Mondal MS Tanaka M Plasma ghrelin levels in lean and obese humans and the effect of glucose on ghrelin secretion. J Clin Endocrinol Metab. (2002) 87:240–4. 10.1210/jcem.87.1.8129 11788653

[78] Cui H López M Rahmouni K. The cellular and molecular bases of leptin and ghrelin resistance in obesity. Nat Rev Endocrinol. (2017) 13:338–51. 10.1038/nrendo.2016.222 28232667 PMC8904083

[79] Peris-Sampedro F Le May MV Stoltenborg I Schéle E Dickson SL. A skeleton in the cupboard in ghrelin research: where are the skinny dwarfs? J Neuroendocrinol. (2021) 33:e13025. 10.1111/jne.13025 34427011

[80] Tschöp M Smiley DL Heiman ML. Ghrelin induces adiposity in rodents. Nature. (2000) 407:908–13. 10.1038/35038090 11057670

[81] Lauritzen ES Jørgensen JOL Møller N Nielsen S Vestergaard ET. Increased lipolysis after infusion of acylated ghrelin: a randomized, double-blinded placebo-controlled trial in hypopituitary patients. Clin Endocrinol. (2020) 00:1–6. 10.1111/cen.14290 32975853

[82] Lindqvist A Shcherbina L Prasad RB Miskelly MG Abels M Martínez-Lopéz JA Ghrelin suppresses insulin secretion in human islets and type 2 diabetes patients have diminished islet ghrelin cell number and lower plasma ghrelin levels. Mol Cell Endocrinol. (2020) 511:110835. 10.1016/j.mce.2020.110835 32371087

[83] Tuero C Becerril S Ezquerro S Neira G Frühbeck G Rodríguez A. Molecular and cellular mechanisms underlying the hepatoprotective role of ghrelin against NAFLD progression. J Physiol Biochem. (2023) 79(4):833–49. 10.1007/s13105-022-00933-1 36417140

[84] Gray SM Page LC Tong J. Ghrelin regulation of glucose metabolism. Peptides. (2018) 100:236–42. 10.1111/jne.12705 29412824 PMC5805851

[85] Zamir I Stoltz Sjöström E van den Berg J Naumburg E Domellöf M. Insulin resistance prior to term age in very low birthweight infants: a prospective study. BMJ Paediatr Open. (2024) 8:e002470. 10.1136/bmjpo-2023-002470 38341196 PMC10862284

[86] Castorina S Barresi V Luca T Privitera G De Geronimo V Lezoche G Gastric ghrelin cells in obese patients are hyperactive. Int J Obes. (2021) 45:184–94. 10.1038/s41366-020-00711-3 33230309

[87] Bellone S Rapa A Vivenza D Castellino N Petri A Bellone J Circulating ghrelin levels as function of gender, pubertal status and adiposity in childhood. J Endocrinol Invest. (2002) 25:RC13-5. 10.1007/BF03344026 12035950

[88] Patro-Małysza J Trojnar M Skórzyńska-Dziduszko KE Kimber-Trojnar Ż Darmochwał-Kolarz D Czuba M Leptin and ghrelin in excessive gestational weight gain-association between mothers and offspring. Int J Mol Sci. (2019) 20:2398. 10.3390/ijms20102398 31096564 PMC6566238

